# Alignment-free analysis of barcode sequences by means of compression-based methods

**DOI:** 10.1186/1471-2105-14-S7-S4

**Published:** 2013-04-22

**Authors:** Massimo La Rosa, Antonino Fiannaca, Riccardo Rizzo, Alfonso Urso

**Affiliations:** 1 ICAR-CNR, National Research Council of Italy, Viale delle Scienze Ed. 11, 90128, Palermo, Italy

## Abstract

**Background:**

The key idea of DNA barcode initiative is to identify, for each group of species belonging to different kingdoms of life, a short DNA sequence that can act as a true taxon barcode. DNA barcode represents a valuable type of information that can be integrated with ecological, genetic, and morphological data in order to obtain a more consistent taxonomy. Recent studies have shown that, for the animal kingdom, the mitochondrial gene cytochrome c oxidase I (COI), about 650 bp long, can be used as a barcode sequence for identification and taxonomic purposes of animals. In the present work we aims at introducing the use of an alignment-free approach in order to make taxonomic analysis of barcode sequences. Our approach is based on the use of two compression-based versions of non-computable Universal Similarity Metric (USM) class of distances. Our purpose is to justify the employ of USM also for the analysis of short DNA barcode sequences, showing how USM is able to correctly extract taxonomic information among those kind of sequences.

**Results:**

We downloaded from Barcode of Life Data System (BOLD) database 30 datasets of barcode sequences belonging to different animal species. We built phylogenetic trees of every dataset, according to compression-based and classic evolutionary methods, and compared them in terms of topology preservation. In the experimental tests, we obtained scores with a percentage of similarity between evolutionary and compression-based trees between 80% and 100% for the most of datasets (94%). Moreover we carried out experimental tests using simulated barcode datasets composed of 100, 150, 200 and 500 sequences, each simulation replicated 25-fold. In this case, mean similarity scores between evolutionary and compression-based trees span between 83% and 99% for all simulated datasets.

**Conclusions:**

In the present work we aims at introducing the use of an alignment-free approach in order to make taxonomic analysis of barcode sequences. Our approach is based on the use of two compression-based versions of non-computable Universal Similarity Metric (USM) class of distances. This way we demonstrate the reliability of compression-based methods even for the analysis of short barcode sequences. Compression-based methods, with their strong theoretical assumptions, may then represent a valid alignment-free and parameter-free approach for barcode studies.

## Background

The use of DNA sequences in order to integrate ecological, morphological and genetic information to improve taxonomic studies of biological species [1] has been carried out since 2003 by Herbert *et al. *[2]. The authors introduced and discussed the need of having DNA sequences as taxon "barcodes". The main purpose was to identify, for each kingdom of life (animals, plants, fungi, and so on) a short DNA fragment that could exploit biodiversity among different species. This way taxonomists can focus above all on discovering new species and describing and fixing existing taxa, leaving identification issues to barcode-based tools [3].

A 648-bp region of the *cytochrome c oxidase I *(COI) gene has been identified as a DNA barcode sequence for the animal kingdom [4]. DNA barcode approach has proven to be useful for the study of biodiversity of very different species, including fishes [5,6], birds [7], bugs [8-10].

The analysis of DNA barcode sequences is usually done by means of clustering methods, like for instance Neighbor Joining (NJ) method [11], that allow to obtain phylogenetic trees (dendograms) of input sequences. Taxonomic studies with DNA barcoding data relies on traditional approaches, that consist of evaluating genetic distances among species in order to perform distance-based clustering analysis [12]. Moreover genetic distances computation needs a preprocessing step, that is sequence alignment, in order to compare corresponding loci. Genetic distances, also called evolutionary distances, are stochastic estimates and they do not define a distance metric [13].

In this work we propose a novel alignment-free approach, for the analysis of DNA barcode data based on information theory concepts. Our aim is to employ Universal Similarity Metric (USM) [14] in order to compute genetic distances among biological species described by DNA barcode sequences. USM represents a class of distance measures based on Kolmogorov complexity [15] and that defines, under some assumptions, a distance metric.

USM is said to be universal because it can be applied for the analysis of data belonging to very different domains: it, in fact, has been used in the field of text and language analysis, image and sound processing [16]. As said earlier, USM is based on Kolmogorov complexity which is, unfortunately, not computable. For this reason, USM needs to be approximated. One of USM's approximation, called Normalized Compression Distance (NCD), has been adopted for the first time for the analysis of biological sequences in [16], where it has been built a coherent phylogenetic tree of 24 species belonging to Eutherian orders considering complete mammalian mtDNA sequences. Another compression-based approximation, the Information-Based Distance (IBD) [17], was applied for the study of whole mitochondrial genome phylogeny. USM and its compression-based approximations have also been used for the analysis of different biological datasets in [18], including protein and genomic (complete mithocondrial genome) sequences. The authors compared phylogenetic trees obtained through USM with gold standard trees using F-measure [19] and Robinson metric [20], obtaining encouraging results about USM use in bioinformatics. NCD has also been adopted for clustering of bacteria considering 16S rRNA gene sequences and topographic representations obtained by means of Self-Organizing Map algorithm [21,22].

Our proposed approach, then, wants to demonstrate that it is possible to apply information theory techniques to the study of short biological sequences for taxonomic and phylogenetic purposes. Genetic distances, obtained through USM's approximations, will be used in order to compute phylogenetic trees of 30 barcode sequence datasets and then those trees will be compared with the ones obtained using traditional bioinformatics approaches depending on sequence-alignment and evolutionary distances computation. The presented results, showing a trees' similarity between 80% and 100%, demonstrates our approach can be adopted for the afore mentioned analysis. In order to further validate our results, we also made experimental tests with simulated barcode datasets, composed of 100, 150, 200 and 500 sequences. For each dataset composition, we considered 25 different barcode datasets, for a total of 100 experiments. The presented results, showing a trees' similarity between 83% and 99% for all simulations, strenghten our findings with real barcode datasets.

In this work, we use USM's compression-based approximations for a deep study and analysis of short DNA barcode sequences. Preliminary results about this topic were presented in [23].

## Methods

**Figure 1:**
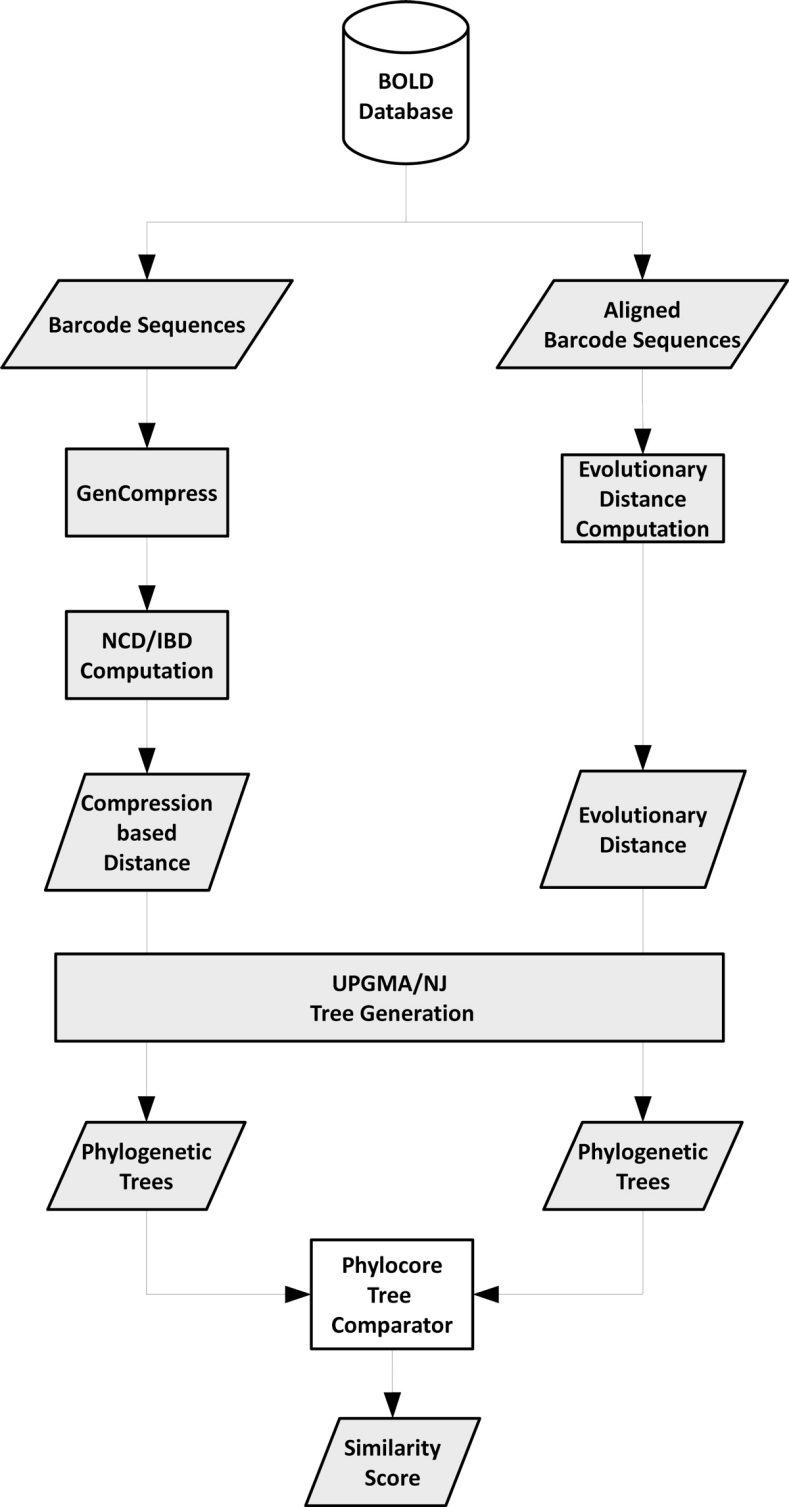
**General flowchart of the proposed comparison approach for real barcode datasets**. Global flowchart of the proposed approach showing all the phases of our experimental setup with real barcode datasets.

The study of application of USM's compression-based approximations to barcode sequences data has been carried out considering both Normalized Compression Distance (NCD) and Information-Based Distance (IBD). Those two distances have been used to compute dissimilarities among species belonging to different kingdoms of life. DNA barcode datasets have been downloaded from Barcode of Life Data System (BOLD) [24], which represents the best source and repository for barcode sequences. In our work we considered 30 datasets of different size and species composition. Using NCD and IBD dissimilarity matrices, we built phylogenetic trees of each of the thirty datasets through two state-of-the-art phylogenetic algorithms, Neighbor Joining and Unweighted Pair Group Method with Arithmetic Mean. Those trees were compared with the ones obtained from five different kinds of evolutionary distances (see next Sections). Figure 1 shows the flowchart of the experimental setup.

In the following subsections a brief explanation of all the employed techniques and algorithms will be provided.

### USM and compression-based distances

Universal Similarity Metric is a class of distance measures defined in terms of Kolmogorov complexity. The Kolmogorov complexity *K*(*x*) of a string *x *is the length of the shortest binary program x* to compute *x *on a universal Turing machine [14,15]. *K*(*x*|*y*) represents the conditional Kolmogorov complexity of two strings, *x *and *y*, and it is defined as the length of the shortest binary program that produces *x *as output, given the input *y *[14,15]. In other terms, *K*(*x*|*y*) is the amount of minimal information needed to generate *x *when *y *is given as input.

The Information Distance (ID) [25] between two objects is then defined as:

(1) ID(x,y)=max{K(x|y),K(y|x)}

It has been shown [25] that ID represents a metric, that means it satisfies the following conditions:

1. ID(*x*, *y*) ≥ 0 (separation axiom);

2. ID(*x*, *y*) = 0 if and only if *x *= *y *(identity axiom);

3. ID(*x*, *y*) = ID(*y*, *x*) (symmetry);

4. ID(*x*, *z*) ≤ ID(*x*, *y*) + ID(*y*, *z*) (triangle inequality).

USM has been presented in [14] and defined as:

(2) $$\text{USM}=\frac{\text{ID}\left(x,y\right)}{\text{max}\;\left\{K\left(x\right),\;K\left(y\right)\right\}}=\frac{\text{max}\;\left\{K\left(x|y\right),\;K\left(y|x\right)\right\}}{\text{max}\;\left\{K\left(x\right),\;K\left(y\right)\right\}}$$

It has been demonstrated [14] that USM is a metric, is normalized (it ranges between 0 an 1) and is universal.

In order to adopt USM as a distance measure, it needs to be approximated since Kolmogorov complexity is not computable. In our work we considered two USM approximations based on data compression: Normalized Compression Distance (NCD) and the Information-Based Distance (IBD) defined in [17]. We chose NCD and IBD because they have been successfully used for the analysis of biological data [16-18,21,22].

NCD and IBD are respectively defined as:

(3) $$\text{NCD}\left(x,y\right)\;=\;\frac{C\left(xy\right)-\text{min}\left\{C\left(x\right),\;C\left(y\right)\right\}}{\text{max}\left\{C\left(x\right),\;C\left(y\right)\right\}}$$

(4) $$\text{IBD}\left(x,y\right)\;=\;1-\frac{C\left(x\right)-C\left(x|y\right)}{C\left(xy\right)}$$

In Eq. (3) and (4), *C*(*x*) is the size, in byte, of the compression version of string *x*; *C*(*xy*) is the size of the compressed version of the concatenation of string *x *and *y*; *C*(*x*|*y*) is the size of the conditional compression of string *x *given string *y*. The basic idea of a string compression algorithm is to find portions of input string that are repeated and to substitute them with a shorter reference. The set of repeated string portions is indicated as "dictionary". Compressing a string *x *given a string *y *means that the compression algorithm builds the dictionary using the string *y *and makes the references on string *x *using that dictionary. This gives a measure of the similarity between the two strings. Both NCD and IBD give better USM approximations if the string are compressed with optimized compression-ratios.

In our experiments, it has been used GenCompress [26] compressor in order to compute both NCD and IBD. GenCompress, in fact, is a Lempel and Ziv dictionary based compressor [27] optimized to work with DNA sequences. If GenCompress is used with generic text strings, as input, it works as a generic ascii-text compressor, without any optimization property.

### Evolutionary distances and phylogenetic trees

Evolutionary distances are distance measures used in order to compute the dissimilarity among genetic sequences [13]. Evolutionary distances are estimates obtained through stochastic methods that take into account many biological phenomena such as convergent substitutions, multiple substitutions per site or retro-mutations. There exist several kinds of evolutionary distance according to the prior assumptions of the stochastic model adopted and their related complexity. The more complex the model, the more accurate and computational expensive the resulting evolutionary distance. In our work, we used five different evolutionary distances, sorted by complexity level, in order to compute phylogenetic trees: Kimura 2-parameter [28], Tajima-Nei [29], Tamura 3-parameter [30] Tamura-Nei [31] and Maximum Composite Likelihood (MCL) [32]. Kimura 2-parameter distance model corrects for multiple hits, taking into account transitional and transversional substitution rates, while assuming that the four nucleotide frequencies are the same and that rates of substitution do not vary among sites. Tajima-Nei distance model derives from the simpler Jukes-Cantor distance [33]and it gives a better estimate of the number of nucleotide substitutions. Tajima-Nei model assumes an equality of substitution rates among sites and between transitional and transversional substitutions. Tamura 3-parameter model corrects for multiple hits, taking into account the differences in transitional and transversional rates and the G+C-content bias. The Tamura-Nei distance with the gamma model corrects for multiple hits, taking into account the different rates of substitution between nucleotides and the inequality of nucleotide frequencies. As for MCL model, a composite likelihood is defined as a sum of log-likelihoods for related estimates. In [32] it is showed that pairwise evolutionary distances and the related parameters are accurately estimated by maximizing the composite likelihood. It is also stated that a complex model had virtually no disadvantage in the composite likelihood method for phylogenetic analyses. In our case, the maximum composite likelihood method is used for describing the sum of log-likelihoods for all pairwise distances estimated by using the Tamura-Nei model. Evolutionary distances were computed using MEGA 5 software [34].

Phylogenetic relationships among biological species are usually inferred by means of phylogenetic trees [35]. In our work we considered the two most popular distance-based algorithms to build phylogenetic trees: Neighbor Joining (NJ) [11] and Unweighted Pair Group Method with Arithmetic Mean (UPGMA) [36]. NJ and UPGMA are said "distance-based" because they need as input a dissimilarity (distance) matrix among elements. Our goal is not to compare the two tree construction methods, but to build and to compare two trees, one with evolutionary distance and the other with compression distance, first using NJ and after using UPGMA.

### Phylogenetic trees comparison algorithms

It is possible to obtain different phylogenetic trees, for the same input dataset, according to the adopted distance measure and/or the used algorithm. That's the reason why there are methods to compute similarity between trees, so that it is possible to understand the shared information content among them. One of the most popular similarity measures between phylogenetic trees is the symmetric distance introduced by Robinson and Foulds [20]. Robinson's metric considers as tree distance the number of "shifts", i.e. edit operations, required to obtain the second tree from the first one (and vice-versa). This approach makes the symmetric distance a "local" similarity algorithm, because it penalizes, in the same way, all the mis-pairings without considering the global clustering results and the tree's topology representing the actual phylogenetic relationships.

For this reason, in our work, we adopted one more recent algorithm for trees' comparison: the PhyloCore algorithm developed by Nye *et al. *[37], that has a different approach from Robinson's one. PhyloCore, in fact, builds an alignment between trees by matching corresponding branches that share the same leaf elements. Each edge (branch) in a phylogenetic tree divides the tree into two subtrees, creating this way a partition of the leaf nodes into two subsets. Each pair of edges between two trees is given a score by comparing the two corresponding partition of leaf elements. Trees partitions with the same leaf nodes represent corresponding clusters and then a similarity in terms of topology and phylogenetic preservation. PhyloCore gives the percentage of topology similarity between trees.

## Results and discussion

In order to extensively test the proposed compression-based approach we used both real and synthetic datasets and compared the results with the ones obtained using the evolutionary distances. In the following subsections we will describe the proposed methodologies and we will discuss the comparison between the two approaches.

### Barcode datasets

We performed our experiments considering real barcode datasets all taken from Barcode Of Life Database (BOLD). Since our purpose was to test the reliability of compression-based distance models, we considered a subset of the whole database. We selected 30 datasets that differ each other on the basis of the type of species (birds, fish, and so on), the number of species, the number of barcode sequences per species (specimens), the sequence length and the sequence quality, expressed in terms of the percentage of sequences with undefined nucleotides, marked with the "N" character. We did not consider all BOLD database because we had no interest in obtaining a phylogenetic tree for all available datasets. It is very important to consider the percentage of sequences containing undefined bases because, as highlighted in Section "Methods", Gen-Compress works as an optimized compressor for DNA sequences only when dealing with string having the four letters A,C,G,T. In all other situations, GenCompress works as a generic ascii text compressor. That means GenCompress will give bad compression ratios for those sequences, and as a consequence NCD and IBD distance (see Eq. (3) and (4)) will not properly approximate USM. Since typical sequence length of COI barcode gene is about 650 bp [4], longer sequences contain information content related to other genes; whereas shorter sequences have incomplete information content. In our study, we then considered as "good" those datasets having a low percentage of sequences with undefined bases and sequences of about the same length (the 650 bp length of typical COI barcode sequence).

The complete list of the barcode datasets of our experiments is summarized in Table 1 and Table 2.

**Table 1:** Barcode datasets.

Dataset Description	Project Code	Taxonomic Description
Small mammal survey in Bakhuis reference sequences	ABSMC	Eutheria

Amazon Fishes	AECI	Actinopterygii

Annotated Genbank Fishes AGF 2006	AGFDO	Perciformes

Annotated Genbank Fishes AGF 2008	AGFSU	Siluriformes

Selected GenBank Bird AGB 2009	AGLUO	Passeriformes

Selected GenBank Bird AGB 2005	AGWEB	Piciformes

Arctiidae - Neotropical fauna PUBLIC records	ARCPU	Lepidoptera

Barcode Accumulation Curves Churchill Parasitoid BAC II	BACX	Hymenoptera

ROM-Bats of Panama	BCUB	Chiroptera

BioLep Sphingidae 1	BLSPA	Lepidoptera

Phaeophyceae Brown Barcode Protocol Proj	BRBP	Phaeophyceae

AMNH Bushmeat Barcoding	BSHMT	Mammalia, Reptilia

Larvae of Chinese Hydropsychidae Part I	CNLVA	Trichoptera

Tetrahymenine Barcoding Project	DLTC	Hymenostomatida

Alaska Crabs	DSALA	Decapoda

Betta of Thailand	DSANA	Perciformes

Actinopterygii of Churchill	DSFCH	Actinopterygii

Fauna Germanica - Lepidoptera Geometridae Others	FBLGO	Lepidoptera

Fauna Germanica - Lepidoptera Macro-Microleps	FBLOT	Lepidoptera

Genbank Fungi - Ascomycota	GBFBA	Ascomycota

Lepidoptera - South American Ennominae Public	GZPSE	Lepidoptera

Ant Diversity in Northern Madagascar	JDWAM	Hymenoptera

JEMU Tephritidae	JTB	Diptera

Trichoptera of Churchill 2005	MHTRI	Trichoptera

Southern New England Leps	MJMSL	Lepidoptera

Onychophora	Onychophora	Onychophora

Plocamium in northern Europe	PLOCE	Plocamiales

Mysids of the Holarctic	RDMYS	Mysida

Birds of Hawaii	SIBHI	Aves

DNA barcodes of marine fishes from the northeast Pacific Ocean and Bering Sea	WXYZ	Actinopterygii

The 30 barcode datasets used in our experimental tests taken from BOLD database

**Table 2:** Barcode datasets description.

DATASET	# Species	# Specimens	% Sequences with undefined bases	Sequence Length
**ABSMC**	46	72	1.3%	650-657

**AECI**	30	30	0.0%	605-679

**AGFDO**	22	22	0.0%	901

**AGFSU**	42	48	2.0%	633-639

**AGLUO**	38	46	2.1%	630

**AGWEB**	33	33	87.0%	900

**ARCPU**	28	52	5.0%	625-658

**BACX**	74	119	2.5%	616-657

**BCUB**	30	108	0.9%	657

**BLSPA**	86	86	4.0%	604-658

**BRBP**	17	106	0.0%	658

**BSHMT**	22	141	5.6%	645

**CNLVA**	33	73	5.0%	625-658

**DLTC**	40	67	1.5%	689-1821

**DSALA**	12	44	11.0%	649-651

**DSANA**	14	274	0.0%	652

**DSFCH**	17	173	3.4%	620-650

**FBLGO**	44	122	2.4%	580-658

**FBLOT**	34	64	3.0%	419-658

**GBFBA**	27	27	7.0%	669

**GZPSE**	23	78	7.7%	601-658

**JDWAM**	103	226	8.8%	620-650

**JTB**	53	225	0.4%	658-899

**MHTRI**	13	108	3.7%	620-650

**MJMSL**	76	198	4.5%	559-658

**Onychophora**	52	210	0.9%	451-884

**PLOCE**	33	102	0.0%	620-660

**RDMYS**	6	37	32.0%	636

**SIBHI**	38	85	0.0%	650-694

**WXYZ**	9	34	3.0%	650-680

The main features of the 30 barcode datasets used in our experimental tests

### Data simulation

In order to test our approach even in case of synthetic data, we simulated some barcode datasets obtained using a generation strategy similar to the one reported in [38,39]. First of all we started by simulating a random ultrametric species tree with Mesquite software (version 2.75, build 564) [40] using the Yule model [41]. We generated four different simulated species trees considering respectively 10, 15, 20 and 50 species, with a total tree depth of 1 million generations. Gene trees were then simulated on the species trees, using the Coalescent package of Mesquite, considering 10 individuals (specimens) per species, obtaining this way gene trees with, respectively, 100, 150, 200 and 500 individuals. Gene trees were simulated using an effective population size of 10000 elements. We finally added noise to the gene trees in order to produce non-ultrametric trees. We considered normally distributed noise with a variance of 0.7 times the original branch length, ad done in [38].

Sequences barcode datasets were simulated, from the gene trees, using the Seq-gen software (version 1.3.3) [42]. We adopted the HKY model of evolution [43], with a transition/transversion ratio of 3, nucleotide frequencies of 0.3 (A), 0.2 (C), 0.2 (G), 0.3 (T), and sequence length of 650 bp, representing the typical COI gene length. For each gene tree, we obtained 25 barcode datasets, resulting in a total of 100 simulated datasets.

### Experimental results

The purpose of the proposed experimental tests is to demonstrate that compression-based distances represent a valid alignment-free approach for the analysis of phylogenetic relationships among short barcode sequences. In Tables 3, 4, 5, 6, 7 there are summarized the similarity scores, obtained using PhyloCore score, among evolutionary based trees and compression based trees of real barcode datasets. More in detail, for every pair of compression-based distances (NCD and IBD) and for every pair of phylogenetic tree inference algorithms (NJ and UPGMA), each table gives the similarity scores according to a reference evolutionary distance model (Kimura 2-parameter, Tamura-Nei and so on).

**Table 3:** Tree similarity score among compression-based trees and evolutionary trees obtained with Kimura 2-parameter distance.

Dataset	PhyloCore			
	NCD		IBD	
	NJ	UPGMA	NJ	UPGMA
**ABSMC**	0.61	0.75	0.59	0.85

**AECI**	0.79	0.86	0.77	0.84

**AGFDO**	0.77	0.89	0.81	0.92

**AGFSU**	0.76	0.73	0.89	0.77

**AGLUO**	0.72	0.80	0.72	0.82

**AGWEB**	0.86	0.84	0.85	0.85

**ARCPU**	0.60	0.78	0.87	0.81

**BACX**	0.76	0.79	0.79	0.80

**BCUB**	0.87	0.92	0.87	0.93

**BLSPA**	0.94	0.87	0.87	0.87

**BRBP**	0.89	0.99	0.82	0.99

**BSHMT**	0.81	0.88	0.74	0.90

**CNLVA**	0.81	0.91	0.88	0.94

**DLTC**	0.88	0.92	0.88	0.92

**DSALA**	0.82	0.89	0.82	0.90

**DSANA**	0.72	0.91	0.63	0.91

**DSFCH**	0.68	0.84	0.54	0.81

**FBLGO**	0.85	0.88	0.85	0.88

**FBLOT**	0.90	0.92	0.90	0.94

**GBFBA**	0.82	0.88	0.79	0.85

**GZPSE**	0.93	0.97	0.95	0.92

**JDWAM**	0.81	0.88	0.80	0.87

**JTB**	0.84	0.84	0.85	0.89

**MHTRI**	0.59	0.90	0.60	0.89

**MJMSL**	0.90	0.97	0.90	0.98

**Onychophora**	0.88	0.91	0.88	0.91

**PLOCE**	0.93	0.95	0.91	0.94

**RDMYS**	0.81	0.89	0.82	0.90

**SIBHI**	0.82	0.88	0.79	0.88

**WXYZ**	0.79	0.92	0.76	0.95

PhyloCore similarity scores of the 30 barcode datasets. The score are obtained comparing compression-based trees, using both NCD and IBD, with evolutionary-based trees, using Kimura 2-parameter distance. The trees were generated through NJ and UPGMA algorithm.

**Table 4:** Tree similarity score among compression-based trees and evolutionary trees obtained with Tajima-Nei distance.

Dataset	PhyloCore			
	NCD		IBD	
	NJ	UPGMA	NJ	UPGMA
**ABSMC**	0.93	0.95	0.95	0.93

**AECI**	0.81	0.87	0.82	0.87

**AGFDO**	0.88	0.92	0.88	0.92

**AGFSU**	0.87	0.83	0.85	0.88

**AGLUO**	0.90	0.97	0.90	0.98

**AGWEB**	0.76	0.73	0.89	0.77

**ARCPU**	0.94	0.87	0.87	0.87

**BACX**	0.76	0.80	0.79	0.80

**BCUB**	0.87	0.89	0.87	0.90

**BLSPA**	0.82	0.88	0.78	0.85

**BRBP**	0.89	1,00	0.82	1,00

**BSHMT**	0.80	0.87	0.74	0.90

**CNLVA**	0.81	0.91	0.87	0.94

**DLTC**	0.78	0.86	0.76	0.85

**DSALA**	0.87	0.91	0.87	0.91

**DSANA**	0.72	0.91	0.63	0.91

**DSFCH**	0.68	0.84	0.54	0.80

**FBLGO**	0.85	0.87	0.84	0.87

**FBLOT**	0.82	0.89	0.83	0.90

**GBFBA**	0.80	0.86	0.85	0.87

**GZPSE**	0.86	0.84	0.85	0.85

**JDWAM**	0.81	0.87	0.80	0.87

**JTB**	0.61	0.75	0.59	0.84

**MHTRI**	0.59	0.89	0.59	0.88

**MJMSL**	0.82	0.88	0.79	0.88

**Onychophora**	0.77	0.89	0.81	0.92

**PLOCE**	0.93	0.95	0.91	0.94

**RDMYS**	0.60	0.72	0.87	0.76

**SIBHI**	0.90	0.93	0.92	0.95

**WXYZ**	0.79	0.89	0.76	0.89

PhyloCore similarity scores of the 30 barcode datasets. The score are obtained comparing compression-based trees, using both NCD and IBD, with evolutionary-based trees, using Tajima-Nei distance. The trees were generated through NJ and UPGMA algorithm.

**Table 5:** Tree similarity score among compression-based trees and evolutionary trees obtained with Tamura 3-parameter distance.

Dataset	PhyloCore			
	NCD		IBD	
	NJ	UPGMA	NJ	UPGMA
**ABSMC**	0.93	0.97	0.95	0.92

**AECI**	0.81	0.89	0.82	0.90

**AGFDO**	0.88	0.92	0.88	0.92

**AGFSU**	0.87	0.83	0.85	0.88

**AGLUO**	0.90	0.97	0.90	0.98

**AGWEB**	0.76	0.73	0.89	0.77

**ARCPU**	0.94	0.87	0.87	0.87

**BACX**	0.76	0.79	0.79	0.80

**BCUB**	0.87	0.91	0.87	0.93

**BLSPA**	0.82	0.88	0.79	0.85

**BRBP**	0.89	0.99	0.82	0.99

**BSHMT**	0.80	0.87	0.74	0.90

**CNLVA**	0.81	0.91	0.88	0.94

**DLTC**	0.81	0.76	0.81	0.74

**DSALA**	0.88	0.91	0.88	0.91

**DSANA**	0.72	0.91	0.63	0.91

**DSFCH**	0.68	0.84	0.54	0.81

**FBLGO**	0.85	0.87	0.85	0.86

**FBLOT**	0.81	0.89	0.82	0.90

**GBFBA**	0.80	0.80	0.85	0.82

**GZPSE**	0.86	0.84	0.85	0.85

**JDWAM**	0.81	0.88	0.80	0.87

**JTB**	0.61	0.74	0.59	0.84

**MHTRI**	0.59	0.89	0.60	0.88

**MJMSL**	0.82	0.88	0.79	0.88

**Onychophora**	0.77	0.89	0.81	0.92

**PLOCE**	0.93	0.95	0.91	0.94

**RDMYS**	0.60	0.74	0.87	0.77

**SIBHI**	0.90	0.92	0.90	0.94

**WXYZ**	0.79	0.78	0.76	0.78

PhyloCore similarity scores of the 30 barcode datasets. The score are obtained comparing compression-based trees, using both NCD and IBD, with evolutionary-based trees, using Tamura 3-parameter distance. The trees were generated through NJ and UPGMA algorithm.

**Table 6:** Tree similarity score among compression-based trees and evolutionary trees obtained with Tamura-Nei distance.

Dataset	PhyloCore			
	NCD		IBD	
	NJ	UPGMA	NJ	UPGMA
**ABSMC**	0.93	0.95	0.95	0.93

**AECI**	0.81	0.87	0.82	0.87

**AGFDO**	0.88	0.92	0.88	0.92

**AGFSU**	0.86	0.84	0.84	0.89

**AGLUO**	0.90	0.84	0.90	0.85

**AGWEB**	0.76	0.73	0.88	0.77

**ARCPU**	0.90	0.87	0.85	0.87

**BACX**	0.76	0.80	0.78	0.80

**BCUB**	0.89	0.90	0.86	0.91

**BLSPA**	0.80	0.86	0.79	0.85

**BRBP**	0.90	0.99	0.84	0.99

**BSHMT**	0.81	0.87	0.73	0.90

**CNLVA**	0.81	0.91	0.87	0.94

**DLTC**	0.81	0.86	0.81	0.85

**DSALA**	0.87	0.91	0.87	0.91

**DSANA**	0.72	0.91	0.63	0.91

**DSFCH**	0.68	0.83	0.54	0.80

**FBLGO**	0.86	0.87	0.84	0.87

**FBLOT**	0.82	0.89	0.84	0.91

**GBFBA**	0.80	0.86	0.85	0.87

**GZPSE**	0.86	0.84	0.85	0.85

**JDWAM**	0.80	0.88	0.79	0.87

**JTB**	0.64	0.75	0.65	0.84

**MHTRI**	0.59	0.89	0.59	0.88

**MJMSL**	0.83	0.89	0.79	0.89

**Onychophora**	0.77	0.89	0.81	0.92

**PLOCE**	0.93	0.95	0.91	0.94

**RDMYS**	0.60	0.74	0.87	0.77

**SIBHI**	0.90	0.92	0.92	0.93

**WXYZ**	0.82	0.79	0.87	0.77

PhyloCore similarity scores of the 30 barcode datasets. The score are obtained comparing compression-based trees, using both NCD and IBD, with evolutionary-based trees, using Tamura-Nei distance. The trees were generated through NJ and UPGMA algorithm.

**Table 7:** Tree similarity score among compression-based trees and evolutionary trees obtained with MCL distance.

Dataset	PhyloCore			
	NCD		IBD	
	NJ	UPGMA	NJ	UPGMA
**ABSMC**	0.95	0.97	0.95	0.92

**AECI**	0.86	0.85	0.87	0.88

**AGFDO**	0.88	0.88	0.88	0.88

**AGFSU**	0.86	0.81	0.85	0.85

**AGLUO**	0.97	0.99	0.97	1,00

**AGWEB**	0.76	0.77	0.89	0.76

**ARCPU**	0.93	0.87	0.88	0.87

**BACX**	0.76	0.79	0.79	0.80

**BCUB**	0.87	0.91	0.87	0.92

**BLSPA**	0.80	0.86	0.77	0.84

**BRBP**	0.89	1,00	0.82	1,00

**BSHMT**	0.81	0.88	0.73	0.90

**CNLVA**	0.81	0.92	0.87	0.95

**DLTC**	0.79	0.86	0.77	0.84

**DSALA**	0.87	0.90	0.87	0.90

**DSANA**	0.75	0.91	0.66	0.91

**DSFCH**	0.68	0.84	0.53	0.80

**FBLGO**	0.84	0.88	0.84	0.88

**FBLOT**	0.81	0.88	0.82	0.90

**GBFBA**	0.80	0.86	0.85	0.87

**GZPSE**	0.84	0.81	0.84	0.82

**JDWAM**	0.81	0.88	0.80	0.87

**JTB**	0.63	0.75	0.64	0.83

**MHTRI**	0.59	0.90	0.58	0.89

**MJMSL**	0.81	0.87	0.78	0.88

**Onychophora**	0.78	0.89	0.82	0.92

**PLOCE**	0.93	0.94	0.91	0.94

**RDMYS**	0.60	0.78	0.87	0.81

**SIBHI**	0.92	0.93	0.91	0.95

**WXYZ**	0.78	0.90	0.73	0.92

PhyloCore similarity scores of the 30 barcode datasets. The score are obtained comparing compression-based trees, using both NCD and IBD, with evolutionary-based trees, using MCL distance. The trees were generated through NJ and UPGMA algorithm.

**Figure 2:**
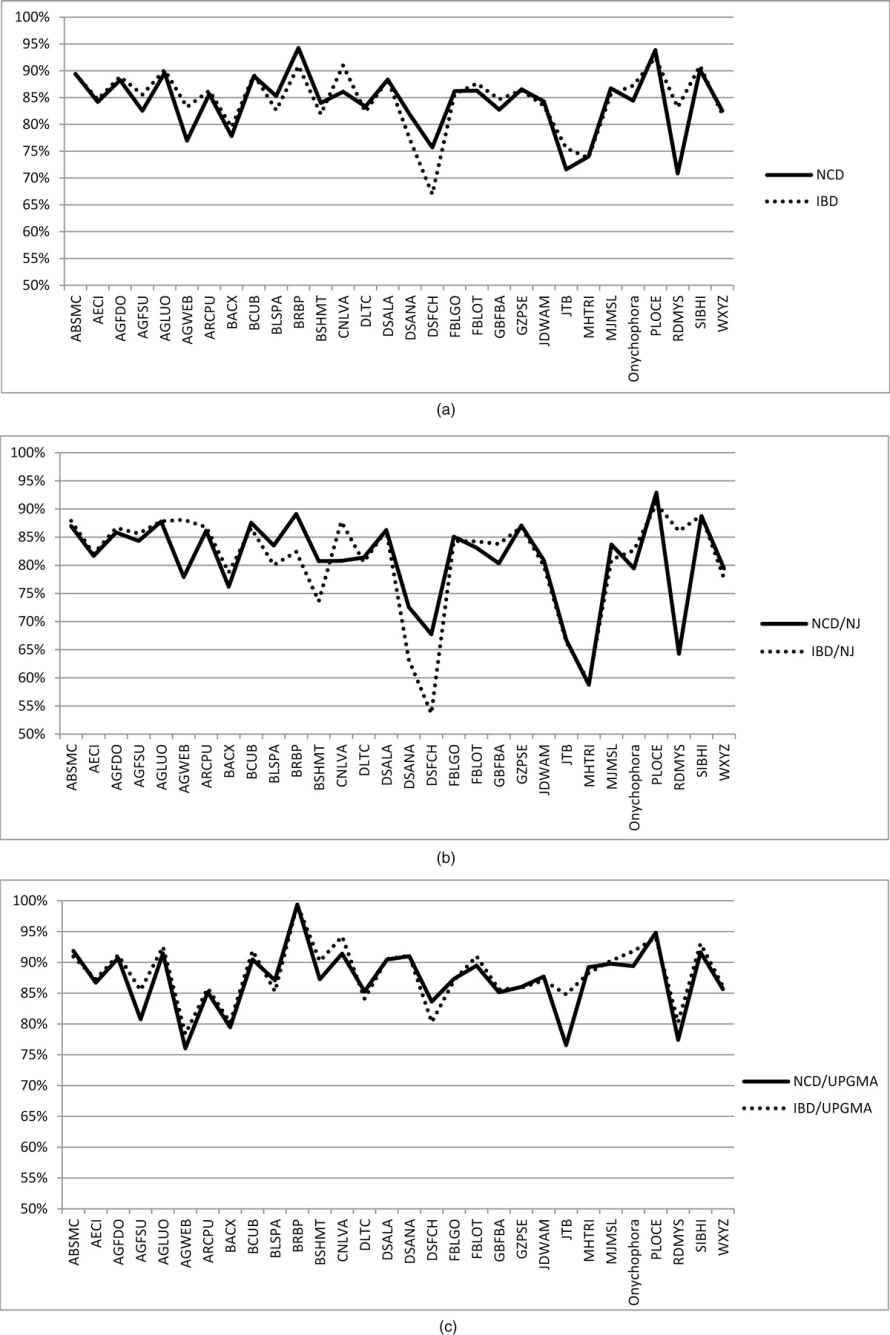
**Mean PhyloCore similarity scores of 30 input datasets**. Mean PhyloCore similarity scores resulting from the comparison among NCD and IBD based trees with the trees obtained from all the five evolutionary distance models. We considered separetely the results obtained using both NJ and UPGMA algorithm(a), only NJ algorithm (b), only UPGMA algorithm (c). The trend curves show NCD and IBD distance models give a quite identical similarity scores in trees' comparison when using UPGMA algorithm for tree inference.

**Figure 3:**
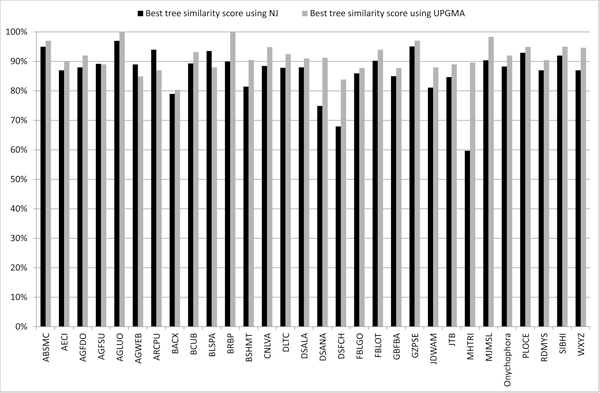
**Histogram of the best similarity scores, for all the evolutionary distance models and input datasets, using NJ and UPGMA algorithm**. In 90% (27/30) of cases, the best similarity scores from comparison among evolutionary based trees and compression based trees are obtained using UPGMA.

Since, in our experiments, we use two kinds of compression-based distances, NCD and IBD, and two different phylogenetic tree inference algorithms, NJ and UPGMA, we are interested in the specific behavior of each distance measure and algorithm. In Figure 2(a) we show the curve trends, related to NCD and IBD methods, representing the PhyloCore similarity mean scores, considering every evolutionary distance model, for the input datasets. The two curves have a similar trend, that is NCD and IBD give very close similarity scores, except for AGWEB, CLNVA, DSFCH and RDMYS datasets. That chart does not give enough information about which compression-based distance produces the most regular results in terms of topology similarity. Our next step was then to check, separately, the similarity scores obtained using the NJ and UPGMA algorithms. In Figure 2(b) and 2(c) we show the trend curves of, respectively, the PhyloCore similarity mean scores, considering every evolutionary distance model and only the NJ algorithm; and the PhyloCore similarity mean scores, considering every evolutionary distance model and only the UPGMA algorithm. From those charts we can state NCD and IBD distance models give quite identical similarity scores in trees' comparison when using UPGMA algorithm for tree inference. Using NJ algorithm, otherwise, we obtain a very unstable trend, with similarity scores generally below than the corresponding scores obtained through UPGMA algorithm. Moreover, in Figure 3 we show in an histogram the highest similarity values, considering all the evolutionary distance models and input datasets, obtained using NJ and UPGMA algorithm. From that chart, we can see that in 90% (27/30) of cases, the best similarity scores from comparison among evolutionary based trees and compression based trees are obtained using UPGMA. That means UPGMA algorithm is the best tree inference algorithm when adopting a compression-based distance models. Looking again at Figure 2(c), the lesser scores, below 80% of similarity, are obtained for AGWEB, JTB, and RDMYS datasets. According to Table 2, AGWEB and RDMYS are the datases with the highest percentage of sequences with undefined bases, respectively 87% and 32%. These low similarity results are then justified by considering the low quality of input datasets, that gave bad compression ratios using GenCompress that in turn produced a bad estimate of NCD and ICD and consequently a wrong phylogenetic tree. As for JTB, its low similarity score is explained considering the different lengths of its sequences, ranging from 658 to 899 bp. As early said in Section "Barcode Datasets", longer sequences contain additional information not related to COI barcode gene and furthermore the spread of sequence length influences NCD and IBD computation (Eq. (3) and (4)).

**Figure 4:**
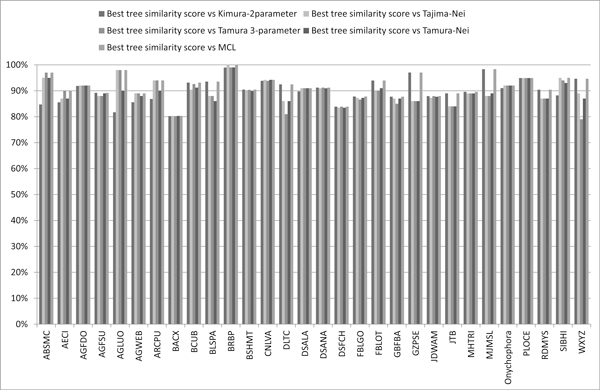
**Histogram of the best Phylocore similarity scores for all input datasets**. For each dataset, it is shown the best similarity score resulting from the pairwise comparison of compression-based trees and the five trees derived from the five evolutionary distance models. The chart shows the highest similarity scores are reached in the comparison among compression-based trees and evolutionary-based trees obtained through MCL distance model.

**Figure 5:**
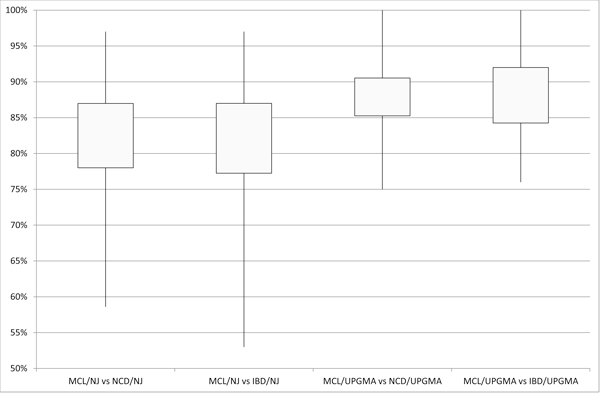
**Boxplot of similarity scores obtained comparing MCL-based trees and compression-based trees using both NJ and UPGMA algorithm**. The best similarity scores, in terms of minimum value, maximum value and mean values, are reached in the comparison between MCL-based trees and compression-based trees, using both NCD and IBD distances, with UPGMA algorithm.

**Figure 6:**
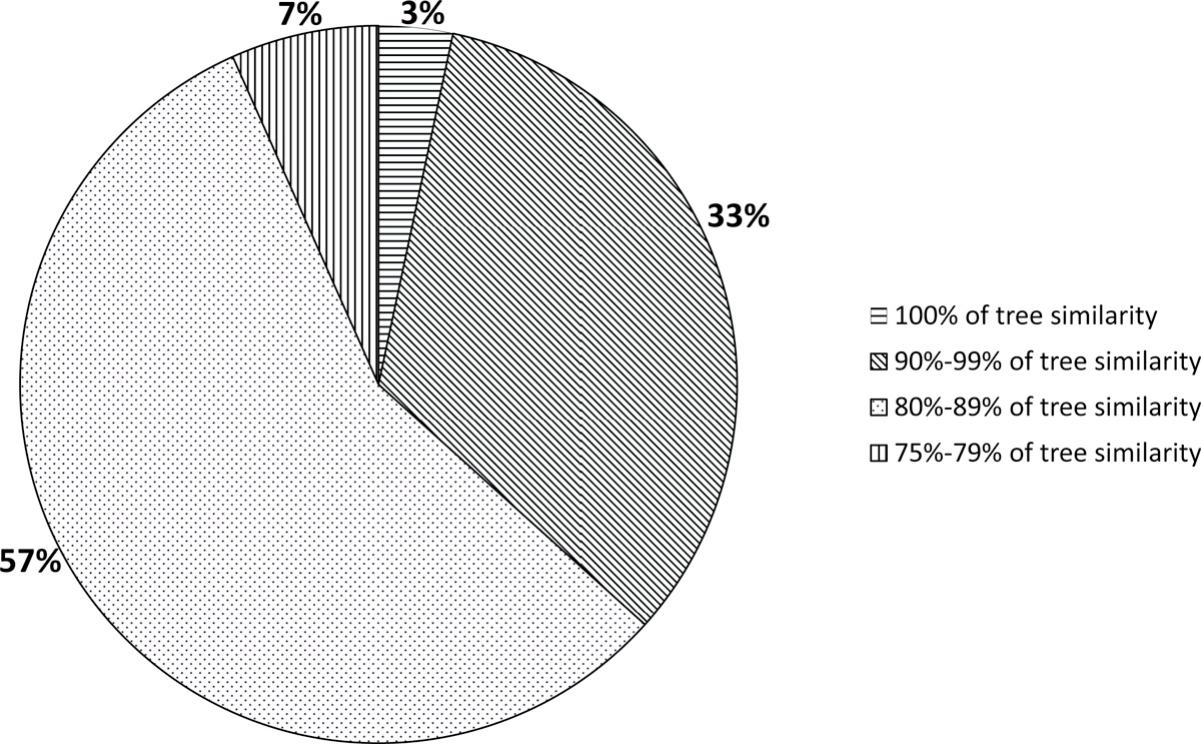
**Piechart summarizing the mean similarity scores among compression-based trees and MCL-based trees obtained using UPGMA algorithm**. From the chart it is shown that in 7% of cases (2/30) we obtain similarity score below 80% (corresponding to AGWEB and JTB datasets); in 57% of cases we have a similarity scores ranging from 80% and 90% (17/30); in 33% of considered datasets (10/30) we obtain a similarity score over 90% and in the 3% of cases (1/30) we reach a 100% of tree similarity.

In order to realize what are the most similar compression-based and evolutionary-based trees, with regards to the evolutionary distance model adopted, we draw the histogram of Figure 4. The histogram is obtained considering the highest similarity values from Tables 3, 4, 5, 6, 7, that is considering both NJ and UPGMA algorithms and both NCD and IBD distance models. The chart in Figure 4 shows the highest similarity scores are reached in the comparison among compression-based trees and evolutionary-based trees obtained through MCL distance model. Moreover in Figure 5 we show the boxplot of similarity scores obtained comparing MCL-based trees and compression-based (NCD and IBD) trees using both NJ and UPGMA algorithm. This chart confirms the best similarity scores, in terms of minimum value, maximum value and mean values, are reached in the comparison between MCL-based trees and compression-based trees using UPGMA algorithm. Finally, in the piechart of Figure 6, we summarize the mean similarity scores for the 30 datasets resulting from the comparison between both compression-based trees and MCL-based trees using UPGMA algorithm. The piechart shows that in 6% of cases (2/30) we obtain similarity score below 80% (corresponding to AGWEB and JTB datasets); in 58% of cases we have a similarity scores ranging from 80% and 90% (17/30); in 33% of considered datasets (10/30) we obtain a similarity score over 90% and in the 3% of cases (1/30) we reach a 100% of tree similarity. It interesting to note that the perfect similarity score (100%) is obtained for BPRP dataset that, as reported in Table 2, represents an ideal barcode dataset, with 658bp sequence lenght and 0% of sequences with undefined bases. As explained in Section "Evolutionary Distances and Phylogenetic Trees", MCL method gives a better estimates of evolutionary distance than the other four distance models, and consequently more accurate phylogenetic trees. From our experimental study we found NCD and IBD compression-based distances,using UPGMA algorithm, build phylogenetic trees that have the best similarity scores with MCL-based trees, which, in turn, give the most accurate phylogenetic relationships.

In order to strengthen our experimental results, we carried out other tests using simulated data, as described in Section "Data Simulation". Results obtained with simulated datasets are summarized in Table 8 and 9. Since we obtained analogous results using both NCD and IBD distance measures, we report only the similarity scores obtained using NCD for sake of simplicity. For each number of input sequences (100, 150, 200, 500), we replicated the simulation 25-fold, for a total of 100 new experiments. Considering all five evolutionary models and the NJ algorithm we evaluated the comparison between compression-based and evolutionary trees, obtaining a very high mean similarity score (83% with a variance between 10^−3 ^and 10^−4^). Using the UPMGMA algorithm the similarity score was even higher with a mean of 99% and a variance between 10^−3 ^and 10^−6 ^.

**Table 8:** Tree similarity score (mean and variance) among compression-based trees and evolutionary trees, obtained with NJ, of simulated datasets.

Simulated Dataset #sequences	Kimura		Tajima-Nei		Tamura		TamuraTamura-Nei		MCL	
	NJ		NJ		NJ		NJ		NJ	
	score	variance	score	variance	score	variance	score	variance	score	variance
100	0.83	4.12E-04	0.83	4.65E-04	0.84	4.77E-04	0.83	4.98E-04	0.83	3.49E-04
150	0.86	8.64E-04	0.86	4.45E-04	0.86	6.44E-04	0.86	4.94E-04	0.86	5.77E-04
200	0.85	1.22E-03	0.85	1.07E-03	0.85	1.23E-03	0.85	9.71E-04	0.84	1.65E-03
500	0.81	1.83E-04	0.81	2.19E-04	0.81	2.23E-04	0.81	2.01E-04	0.81	2.76E-04

PhyloCore similarity scores of the simulated datasets. According to the number of barcode sequences, each simulation was replicated 25-fold, for a total of 100 simulated dataset. The scores (mean and variance) are obtained comparing compression-based trees, using NCD, with evolutionary-based trees obtained through all five evolutionary models. The trees were generated using NJ algorithm.

**Table 9:** Tree similarity score (mean and variance) among compression-based trees and evolutionary trees, obtained with UPGMA, of simulated datasets.

Simulated Dataset #sequences	Kimura		Tajima-Nei		Tamura		TamuraTamura-Nei		MCL	
	UPGMA		UPGMA		UPGMA		UPGMA		UPGMA	
	score	variance	score	variance	score	variance	score	variance	score	variance
100	0.99	8.22E-05	0.99	1.02E-04	0.99	7.23E-05	0.99	6.60E-05	0.99	4.80E-05
150	0.99	7.07E-06	0.99	4.43E-05	0.99	1.42E-05	0.97	4.20E-03	0.99	6.25E-05
200	0.98	1.32E-04	0.98	1.17E-04	0.98	1.28E-04	0.98	9.63E-05	0.98	6.61E-05
500	0.98	2.48E-05	0.98	4.22E-05	0.98	3.94E-05	0.98	4.32E-05	0.98	3.11E-05

PhyloCore similarity scores of the simulated datasets. According to the number of barcode sequences, each simulation was replicated 25-fold, for a total of 100 simulated dataset. The scores (mean and variance) are obtained comparing compression-based trees, using NCD, with evolutionary-based trees obtained through all five evolutionary models. The trees were generated using UPGMA algorithm.

We can state, then, that our proposed approach is very reliable using simulated data and robust enough to be applied with real barcode datasets.

### Speed evaluation

**Figure 7:**
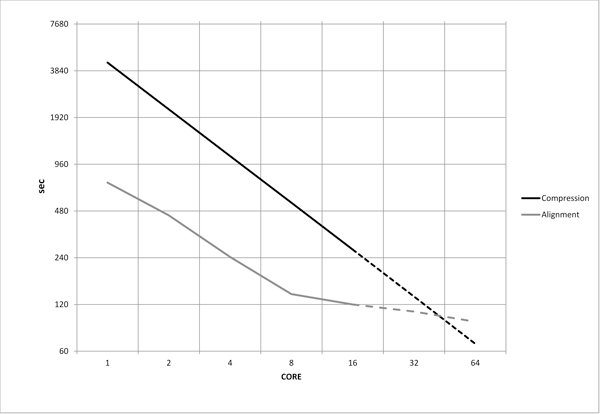
**Execution times, in ***log*_2 _**base, of compression and alignment for dataset of 500 sequences versus the number of processing cores**. The chart, in *log*_2 _base, shows real (solid line) and estimated (dashed line) execution times of both compression and alignment for barcode dataset of 500 sequences. Compression-based approach overcomes alignment approach using a multicore system after 32 cores.

In order to compare the processing time of the proposed algorithm with the speed of evolutionary distance methods, we performed additional experiments. It is possible to notice that the compression-based distance can be calculated separately for each sequence versus all the other, so that, in principle we can calculate all the distance running all the programs at the same time (one program for each sequence running on one processor core), this makes the compression-based method intrinsically parallel. If we want to compare the performance of the proposed method to the one using the alignment distance, we have to take into account a parallel version of the alignment algorithm. We used the algorithm described in [44], that exploits the multi-core processor and becomes faster each time a processor core is available. In this algorithm the speed increment decreases in non-linear way each time we double the number of cores. On the other hand, as said above, in the compression-based distance method the speed increment is constant and each time we double the number of cores, the speed doubles. For this reason if we compare the running time of the two methods in term of number of cores we will find a trade-off point. Experiments for evaluation of running times were carried out using a multicore system up to 16 cores. We tested the execution times of both compression and alignment for barcode dataset of 500 sequences versus the number of cores. Running times are summarized in Figure 7, that shows real (solid line) and estimated (dashed line) times in *log*_2 _base. Compression-based approach overcomes alignment approach using a multicore system after 32 cores.

## Conclusions

In this paper we presented a novel alignment-free approach for the study of barcode genetic sequences. We used two compression-based approximations of USM, namely NCD and IBD, for reconstructing phylogenetic trees of short barcode sequences. In previous works, in fact, compression-based distances were used only for the analysis of whole mithocondrial genomes. We tested our approach considering 30 barcode datasets, of different size and belonging to different species, and 100 simulated datasets composed of different number of sequences (100, 150, 200, 400). Compression-based trees, obtained from NCD and IBD distances, were compared with evolutionary-based trees derived using five evolutionary distance models: Kimura 2-parameter, Tajima-Nei, Tamura 3-parameter, Tamura-Nei and MCL. Trees were obtained using NJ and UPGMA algorithms. Our experimental tests demonstrated that using NCD and IBD compression-based distances we were able to obtain phylogenetic trees quite similar to evolutionary-based trees, with similarity scores ranging from 80% to 100%. More in detail, the highset similarity scores were reached comparing compression-based trees with MCL-based trees using UPGMA algorithm, with no substantial differences between NCD and IBD. MCL provides a better esitmates of evolutionary distance, and as a consequence more accurate phylogenetic trees, than the remaining considered methods. As for simulated data, our experimental trials show very stable results with regards to the number of input sequences and evolutionary model considered, with similarity scores spanning from 83%, using NJ algorithm, and 99%, using UPGMA algorithm. NCD and IBD compression distance models represent a sound alignment-free and parameter-independent approach, based on strong theoretical assumptions. Using these models it is possible to obtain very reliable phylogenetic trees and they are a valid tool for the analysis of barcode sequences.

## Competing interests

The authors declare that there are no competing interests.

## Authors' contributions

MLR: project conception, implementation, experimental tests, writing, assessment, discussions. AF: project conception, writing, assessment, discussions. RR: project conception, discussions, writing. AU: project conception, discussions, writing, funding. All authors read and approved the final manuscript.

## Declarations

The publication costs for this article were funded by the CNR Interomics Flagship Project "- Development of an integrated platform for the application of "omic" sciences to biomarker definition and theranostic, predictive and diagnostic profiles".

This article has been published as part of *BMC Bioinformatics *Volume 14 Supplement 7, 2013: Italian Society of Bioinformatics (BITS): Annual Meeting 2012. The full contents of the supplement are available online at http://www.biomedcentral.com/bmcbioinformatics/supplements/14/S7
